# CircATRNL1 and circZNF608 Inhibit Ovarian Cancer by Sequestering miR-152-5p and Encoding Protein

**DOI:** 10.3389/fgene.2022.784089

**Published:** 2022-02-23

**Authors:** Mengmeng Lyu, Xiujuan Li, Yang Shen, Jin Lu, Lihua Zhang, Shanliang Zhong, Jinhua Wang

**Affiliations:** ^1^ Department of Gynecologic Oncology, The Affiliated Cancer Hospital of Nanjing Medical University and Jiangsu Cancer Hospital and Jiangsu Institute of Cancer Research, Nanjing, China; ^2^ Department of General Surgery, The Affiliated Cancer Hospital of Nanjing Medical University and Jiangsu Cancer Hospital and Jiangsu Institute of Cancer Research, Nanjing, China; ^3^ Center of Clinical Laboratory Science, The Affiliated Cancer Hospital of Nanjing Medical University and Jiangsu Cancer Hospital and Jiangsu Institute of Cancer Research, Nanjing, China

**Keywords:** circular RNA, protein, ovarian cancer, translation, peptide

## Abstract

**Background:** CircRNAs have been found to be involved in the pathogenesis of various diseases. We aimed to explore the roles of circRNAs in ovarian cancer.

**Methods:** The expression levels of circRNAs in ovarian cancer and normal ovarian tissues were analyzed using RNA sequencing. Fluorescent *in situ* hybridization (FISH), proliferation assays and transwell assays were used to assess the effects of circRNAs on ovarian cancer.

**Results:** CircATRNL1 and circZNF608 were downregulated in 20 ovarian cancer tissues compared to normal tissues. CircATRNL1 and circZNF608 are mainly located in the cytoplasm of ovarian cancer cells, and circATRNL1 is a highly conserved circRNA. The overexpression of circATRNL1 and circZNF608 inhibits the proliferation and invasion of ovarian cancer cells. We predicted miRNA–circRNA interactions for circZNF608 and circATRNL1 and obtained 63 interactions. However, a luciferase reporter assay showed that only miR-152-5p was sequestered by circZNF608. Bioinformatics analysis and experiments indicated that circATRNL1 contains an internal ribosome entry site and an open reading frame encoding a 131 aa protein.

**Conclusion:** In conclusion, circATRNL1 and circZNF608 are two downregulated circRNAs in ovarian cancer and work as tumor suppressors. CircZNF608 may exert antitumor activity in ovarian cancer by binding miR-152-5p, and circATRNL1 may encode a 131 aa protein.

## Introduction

Ovarian cancer is the seventh most prevalent female cancer worldwide (Vafadar et al., 2020). Ovarian cancer is usually associated with silent and unclear symptoms, which makes it a difficult disease to diagnose, and it is usually diagnosed in advanced stages (Jayson et al., 2014). Standard treatment options for ovarian cancer include surgery and chemotherapy (Stewart et al., 2019). However, current treatments are unable to cure advanced ovarian cancer. Therefore, early diagnosis represents the best hope for mortality reduction and long-term disease control (Rauh-Hain et al., 2011). The lack of a powerful biomarker for early diagnosis is the main obstacle. New biomarkers and new mechanisms are needed to help manage patients with ovarian cancer.

Recently, circular RNAs (circRNAs) have been shown to exhibit biomarker potential due to their abundance, specificity and stability (Shafabakhsh et al., 2019). Compared to linear RNAs, which feature terminal 5′ caps and 3′ tails, circRNAs have a covalently closed loop structure without 5′ to 3′ polarity and a polyadenylated tail (Zhong et al., 2018a). Therefore, on account of the unique structure, circRNAs were initially underestimated. With the emergence of advanced technology, an increasing number of circRNAs have been identified from different cell lines and species (Zhong et al., 2019). CircRNAs have been found to be involved in the pathogenesis of various diseases, including diabetes (Xu et al., 2015), cardiovascular diseases (Wang et al., 2016), Alzheimer’s disease (Dube et al., 2019) and cancers (Zhong et al., 2018a; Zhang et al., 2019b; Wang et al., 2019; Zhou et al., 2019; Verduci et al., 2021). For example, FECR1, a circRNA derived from exons 2, 3, and 4 of the FLI1 gene, could bind to the promoter of FLI1 and then activate FLI1 by inducing DNA hypomethylation in CpG islands of the promoter, thus promoting the metastasis of breast cancer (Chen et al., 2018). Several studies have explored the roles of circRNA in ovarian cancer (Yang et al., 2020). Gan et al. found that circMUC16 had a higher expression level in ovarian cancer tissues than in healthy ovarian tissues, and its expression level was correlated with the stage and grade of ovarian cancer; the authors further demonstrated that circMUC16 promotes autophagy of epithelial ovarian cancer *via* interaction with ATG13 and miR-199a (Gan et al., 2020). In the present study, we profiled circRNA expression in 3 paired ovarian cancer tissues and normal ovarian tissues and identified two anticancer circRNAs.

## Materials and Methods

### Specimens

We recruited 20 patients with stage III-IV high-grade serous ovarian cancer from the Affiliated Cancer Hospital of Nanjing Medical University. The patients were aged from 50 to 65, born in China, lived in the province of Jiangsu and did not receive any anticancer treatment before surgery. Ovarian cancer tissues and paired normal ovarian tissues were collected, flash frozen in liquid nitrogen, and stored at −80°C. The ethics committee of The Affiliated Cancer Hospital of Nanjing Medical University approved the study protocol, and informed consent was obtained from all the patients.

### Cell Culture

Ovarian cancer cell lines (A2780, SKOV3, and SW626) and a normal ovarian epithelial cell line (IOSE80) were purchased from Shanghai Guan & Dao Biological Engineering Co., Ltd. The identities of the cell lines were authenticated by short-tandem-repeat (STR) profiling. All four cell lines were cultured in DMEM (Keygen Biotech, Nanjing, China) with 10% fetal bovine serum, 100 units/ml penicillin, and 100 μg/ml streptomycin.

### Profiling of circRNAs

We randomly selected 3 paired ovarian cancer tissues and normal ovarian tissues from the 20 paired samples for circRNA profiling. CircRNAs were profiled using the RNA-seq technique by Gene Denovo Biotechnology Co. (Guangzhou, China). Briefly, total RNA was extracted and then treated with Ribonuclease R (RNase R) to degrade linear RNAs. A strand-specific library was constructed using a VAHTS Total RNAseq (H/M/R) Library Prep Kit (Vazyme, Nanjing, China). After removing ribosomal RNAs, the enriched circRNAs were fragmented into short fragments and reverse transcribed into cDNA. Second-strand cDNA was synthesized. The cDNA fragments were purified, end repaired, poly(A) added, and ligated to Illumina sequencing adapters. The second-strand cDNA was digested with uracil-N-glycosylase (UNG). The digested products were purified, PCR amplified, and sequenced using an Illumina Novaseq60000. The raw RNA-seq data have been submitted to the Gene Expression Omnibus (GEO) database (accession number GSE192410).

After obtaining clean reads and removing ribosome RNA (rRNA)-mapped reads, the remaining reads were mapped to the reference genome. Then, the unmapped reads were collected for circRNA identification using find_circ (version 1) (Memczak et al., 2013). Finally, we filtered raw read counts of circRNAs and kept only the genes with a nonzero read count in 3 or more samples and then used DESeq2 (version 1.32) (Love et al., 2014) in the statistical program R (version 4.0.1) to normalize the read counts. Differentially expressed circRNAs were identified based on a fold change of at least 2 and an adjusted *p* value <0.05.

### Real-Time Quantitative PCR

CircPrimer (Zhong et al., 2018b) was used to annotate circRNAs and design primers for circRNAs. We performed reverse transcription and RT–qPCR as in our previous study (Zhong et al., 2018a; Zhou et al., 2020). The expression levels of genes were detected as described in our previous report (Wang et al., 2020b). β-action was used as a reference gene. All samples were analyzed in duplicate for each circRNA.

### Transfection Experiment

Plasmids expressing circATRNL1 (p-circATRNL1) and circZNF608 (p-circZNF608) as well as control plasmids (p-control) were purchased from HanBio Biotechnology (Hanbio, Shanghai, China). Cells in logarithmic phase were harvested and seeded at 4 × 10^5^ cells per well into a 6-well plate. After incubation for 24 h, the cells were transfected using Lipofectamine 3000 (Invitrogen, Carlsbad, CA, USA) following the manufacturer’s instructions.

### Fluorescent *In Situ* Hybridization

The FISH probes and Ribo™ fluorescent *in situ* hybridization Kit were purchased from Guangzhou RiboBio Co., Ltd. (RiboBio, Guangzhou, China). *In situ* hybridization was conducted as described previously (Zhong et al., 2018a; Zhou et al., 2020).

### CCK8 Proliferation Assay

After transfection for 24 h, 4,000 cells were seeded per well (96-well plate) in the presence of 200 μL of complete medium. The cells were divided into four groups. After cell attachment, the medium of the first group was removed, 100 μL CCK8 was added, the cells were incubated at 37°C for 1 h. Then, the absorbance was measured at 450 nm. The other three groups were detected at 24, 48 and 72 h, respectively.

### Transwell Migration and Invasion Assays

Transwell migration and invasion assays were conducted as described previously (Zhou et al., 2020). For cell migration, 4 × 10^4^ cells were seeded into the upper chamber of transwell assay inserts (pore size of 8 mm; Corning) containing 200 μL DMEM medium without serum. We added 600 μL DMEM with 20% fetal bovine serum to the lower chamber of 24-well culture plates. After incubation for 48 h, the cells on the filter surface were fixed with 4% paraformaldehyde, stained with crystal violet and imaged using a microscope (Carl Zeiss). For cell invasion, serum-free DMEM containing 100 μL mixture (1:8) of Matrigel (Corning, NY, USA) was coated on the upper transwell chamber before seeding cells. After allowing 4 h for the Matrigel to solidify, the subsequent experimental steps were similar to those in the migration assay.

### circRNA-miRNA Interaction Prediction

We used circMir (www.bio-inf.cn/circmir) to predict miRNA–circRNA interactions. CircMir is a homemade software that runs miRanda (August 2010 release) and RNAhybrid (version 2.1.2) to obtain miRNA–circRNA interactions. Miranda software was set using the following default parameters: gap extend penalty, −4.0; gap open penalty, −9.0; energy threshold, 1.0 kcal/mol; score threshold, 140.0; and scaling parameter, 4.0. The following parameters were used for RNAhybrid: perfect miRNA seed complementarity with circRNA sequence, miRNA:circRNA binding energy < −26 kcal/mol and *p* value <0.05. Only the circRNA–miRNA interactions predicted by both miRanda and RNAhybrid were kept for further study.

### Luciferase Reporter Assay

MiR mimics and negative control mimics (mimics-NC) were purchased from Guangzhou RiboBio Co., Ltd. (RiboBio, Guangzhou, China). The entire sequences of circZNF608 and circATRNL1 were cloned into the downstream region of the humanized form of Renilla luciferase (hRLuc) of the pmiR-RB-REPORT vector (RiboBio, Guangzhou, China), denoted circZNF608/vector-wd and circATRNL1/vector-wd.

To screen the miRNAs binding to the two circRNAs, miR mimics or negative control mimics (mimics-NC) were cotransfected with the vectors into cells. After 24 h, the luciferase activity was assessed using a dual-luciferase reporter gene assay kit (Beyotime Biotechnology, Shanghai, China). Because we found that only miR-152-5p inhibited the luciferase activity of circZNF608/vector-wd by more than 50%, we further mutated the miR-152-5p binding site from circZNF608/vector-wd to construct circZNF608/vector-mut. Then, mimics-NC or miR-152-5p mimics were cotransfected with circZNF608/vector-wd or circZNF608/vector-mut into cells, and luciferase activity was assessed.

### Statistical Analysis

R software (version 4.0.1) was used to perform all statistical analyses. RT–qPCR results were assessed using the Wilcoxon rank test, paired test for patients and unpaired test for cells. Other data were evaluated using Student’s t test and one-way analysis of variance. A *p* value <0.05 was considered statistically significant.

## Results

### Identification and Characteristics of circZNF608 and circATRNL1

**FIGURE 1:**
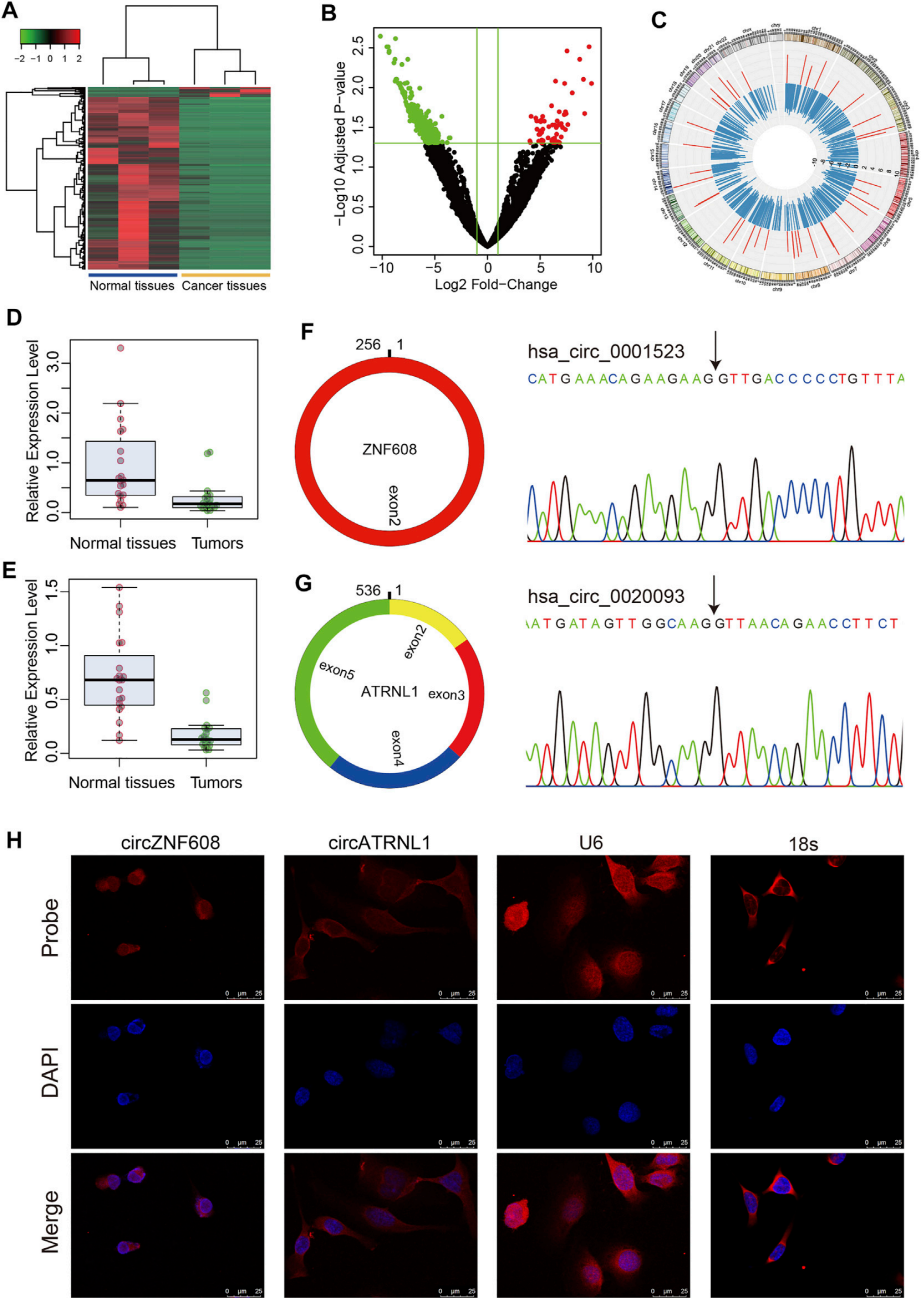
Characteristics of circZNF608 and circATRNL1. **(A)** Heatmap of differentially expressed circRNAs in ovarian cancer tissues compared with normal ovarian tissues. **(B)** Volcano plot of circRNAs in RNA-seq data. Green dots: downregulated circRNAs; red dots: upregulated circRNAs. **(C)** Circos diagram illustrating the fold change of differentially expressed circRNAs in RNA-seq data. The red bar indicates log2 (fold change) of upregulated circRNAs, and the blue bar indicates downregulated circRNAs. **(D)** circZNF608 and **(E)** circATRNL1 were downregulated in ovarian cancer tissues compared with normal ovarian tissues (*p* < 0.001). **(F)** and **(G)** The back-splice junction of circZNF608 and circATRNL1 was confirmed by sequencing the PCR products from the RNase R-treated RNA. **(H)** RNA fluorescence *in situ* hybridization for circZNF608 and circATRNL1. Nuclei were stained with 4,6-DAPI.

We profiled 3 paired ovarian cancer tissues and normal ovarian tissues using RNA-seq, and the raw read counts are presented in Supplementary Table S1. We identified 844 differentially expressed circRNAs in ovarian cancer tissues compared to normal ovarian tissues, including 45 upregulated circRNAs and 799 downregulated circRNAs (Supplementary Table S2, Figures 1A–C).

According to the fold change and normalized read counts, we selected 10 upregulated circRNAs and 10 downregulated circRNAs for RT–qPCR validation using cell lines. The 20 circRNAs can be found in Supplementary Table S2 and are labeled in bold font. Among the 20 circRNAs, 3 circRNAs [circZNF608 (hsa_circ_0001523), circATRNL1 (hsa_circ_0020093) and circATXN10 (hsa_circ_0001246)] were differentially expressed in cell lines. Two (circATRNL1 and circATXN10) of the three circRNAs showed the same trend as RNA-seq, and one (circZNF608) showed the reverse trend. We further validated these three circRNAs in 20 paired tissues. All three circRNAs were downregulated in ovarian cancer tissues compared with normal ovarian tissues (Figure 1D for circZNF608 and Figure 1E for circATRNL1). Because we failed to overexpress hsa_circ_0001246 using plasmids, we focused on the other two circRNAs in the following studies.

We analyzed circZNF608 and circATRNL1 using circPrimer 2.0. CircZNF608 is located at chr5:124701013-124701269- and is derived from exon 2 of the ZNF608 gene with a spliced length of 256 nt (Figure 1F). CircATRNL1 is derived from exons 2–5 of the ATRNL1 gene with a spliced length of 536 nt (Figure 1G). CircATRNL1 is a highly conserved circRNA located at chr10: 115120184–115129535+ in the human genome and chr19: 57703574–57717118+ in the mouse genome (mmu_circ_0007920) (Supplementary Figure S1). The existence of circZNF608 and circATRNL1 was confirmed by Sanger sequencing (Figures 1F,G). FISH showed that circZNF608 and circATRNL1 are mainly located in the cytoplasm of ovarian cancer cells (Figure 1H).

### Overexpression of circZNF608 and circATRNL1 Inhibits Proliferation, Migration and Invasion

**FIGURE 2:**
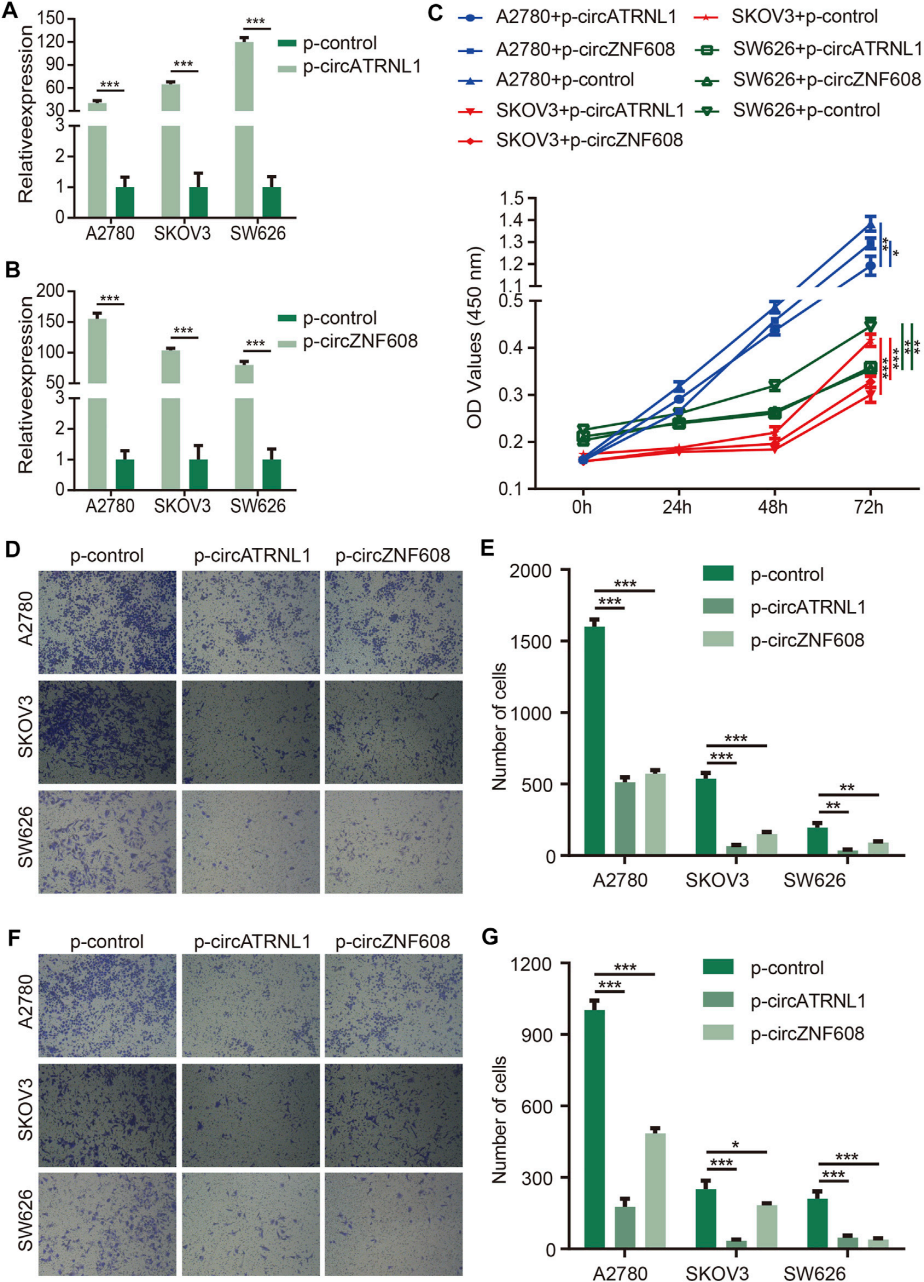
circATRNL1 and circZNF608 overexpression inhibited ovarian cancer cell proliferation and invasion. **(A)** circATRNL1 was overexpressed after transfecting p-circATRNL1. **(B)** circZNF608 was overexpressed after transfecting p-circZNF608. **(C)** circATRNL1 and circZNF608 inhibited the proliferation of ovarian cancer cells. **(D)** Representative pictures of the transwell migration assay. **(E)** circATRNL1 and circZNF608 decreased the migration of ovarian cancer cells. **(F)** Representative pictures of the transwell invasion assay. **(G)** circATRNL1 and circZNF608 decreased the invasion of ovarian cancer cells. p-circATRNL1, plasmids expressing circATRNL1; p-circZNF608, plasmids expressing circZNF608, p-control, control plasmids; *p < 0.05; **p < 0.01; and ***p < 0.001.

We explored the effects of circZNF608 and circATRNL1 on ovarian cancer cells. We transfected p-control, p-circATRNL1, and p-circZNF608 into ovarian cancer cell lines, detected the expression of circATRNL1 and circZNF608, and found that the cells transfected with p-circATRNL1 and p-circZNF608 had higher expression levels of circATRNL1 and circZNF608 than control cells (Figures 2A,B). The CCK8 proliferation assay showed that both circATRNL1 and circZNF608 could inhibit the proliferation of all three ovarian cancer cell lines (Figure 2C). The transwell migration assay indicated that higher expression of circATRNL1 and circZNF608 was associated with lower migration rates (Figures 2D,E). We further explored circATRNL1 and circZNF608 on invasion and found that the overexpression of circATRNL1 and circZNF608 inhibited the invasion of ovarian cancer cell lines (Figures 2F,G).

### CircZNF608 Acts as a miRNA Sponge for miR-152-5p

**FIGURE 3:**
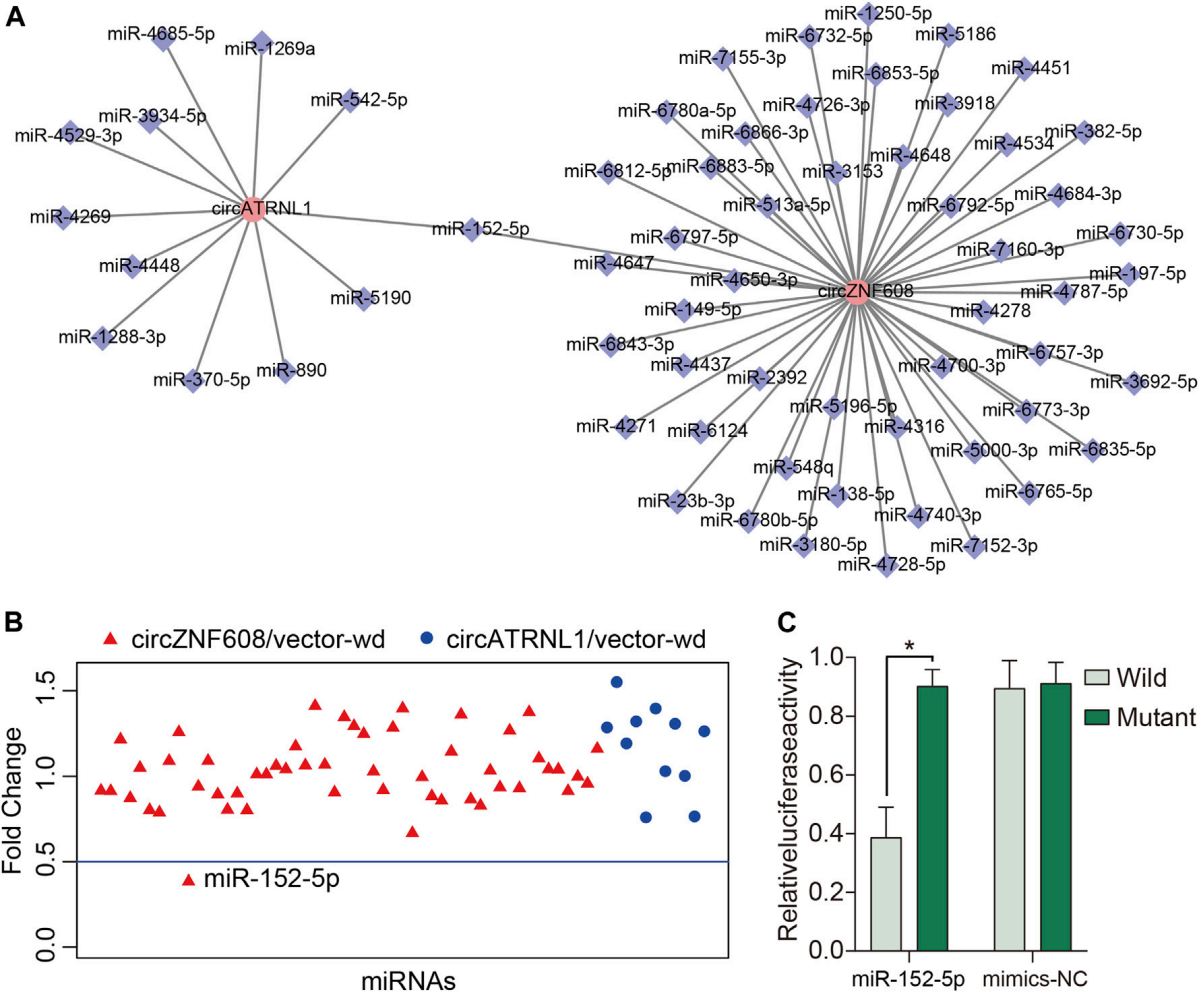
circZNF608 serves as a sponge for miR-152-5p in ovarian cancer cells. **(A)** The 63 predicted miRNA–circRNA interactions for circZNF608 and circATRNL1. **(B)** Luciferase reporter assay of the 63 miRNA–circRNA interactions to identify miRNAs that were able to bind to the circZNF608 or circATRNL1 sequence. Only miR-152-5p inhibited luciferase activity by more than 50%. **(C)** Luciferase reporter assay for the luciferase activity of wild-type and mutant circATRNL1 vectors cotransfected with miR-152-5p mimics or mimic-NC (negative control of mimics).

Studies have reported that the subcellular localization of a circRNA is closely correlated with its function (Panda et al., 2017; Qu et al., 2018). CircRNAs that are mainly located in the cytoplasm may act as miRNA sponges to bind miRNAs (Qu et al., 2018). Because circZNF608 and circATRNL1 are predominantly located in the cytoplasm of ovarian cancer cells, we predicted miRNA–circRNA interactions for circZNF608 and circATRNL1 and obtained 63 interactions (Figure 3A). We screened the miRNAs that bind to circZNF608 and circATRNL1 using a luciferase reporter assay and found that only miR-152-5p inhibited the luciferase activity of circZNF608/vector-wd by more than 50% (Figure 3B). We further mutated the miR-152-5p binding site from circZNF608/vector-wd and constructed circZNF608/vector-mut. CircZNF608/vector-wd or circZNF608/vector-mut was cotransfected with miR-152-5p mimics or mimics-NC, and a luciferase reporter assay showed that miR-152-5p could inhibit the luciferase activity of circZNF608/vector-wd significantly compared to circZNF608/vector-Mut, and mimics-NC had no effect on luciferase activity, suggesting that circZNF608 may exert its anticancer effects by sequestering miR-152-5p (Figure 3C). We further detected the expression of miR-152-5p in the 20 paired ovarian cancer tissues and normal ovarian tissues and failed to find a difference between the two groups (Supplementary Figure S2), suggesting that circZNF608 binds miR-152-5p but does not influence its expression.

### CircATRNL1 Encodes a Protein

**FIGURE 4:**
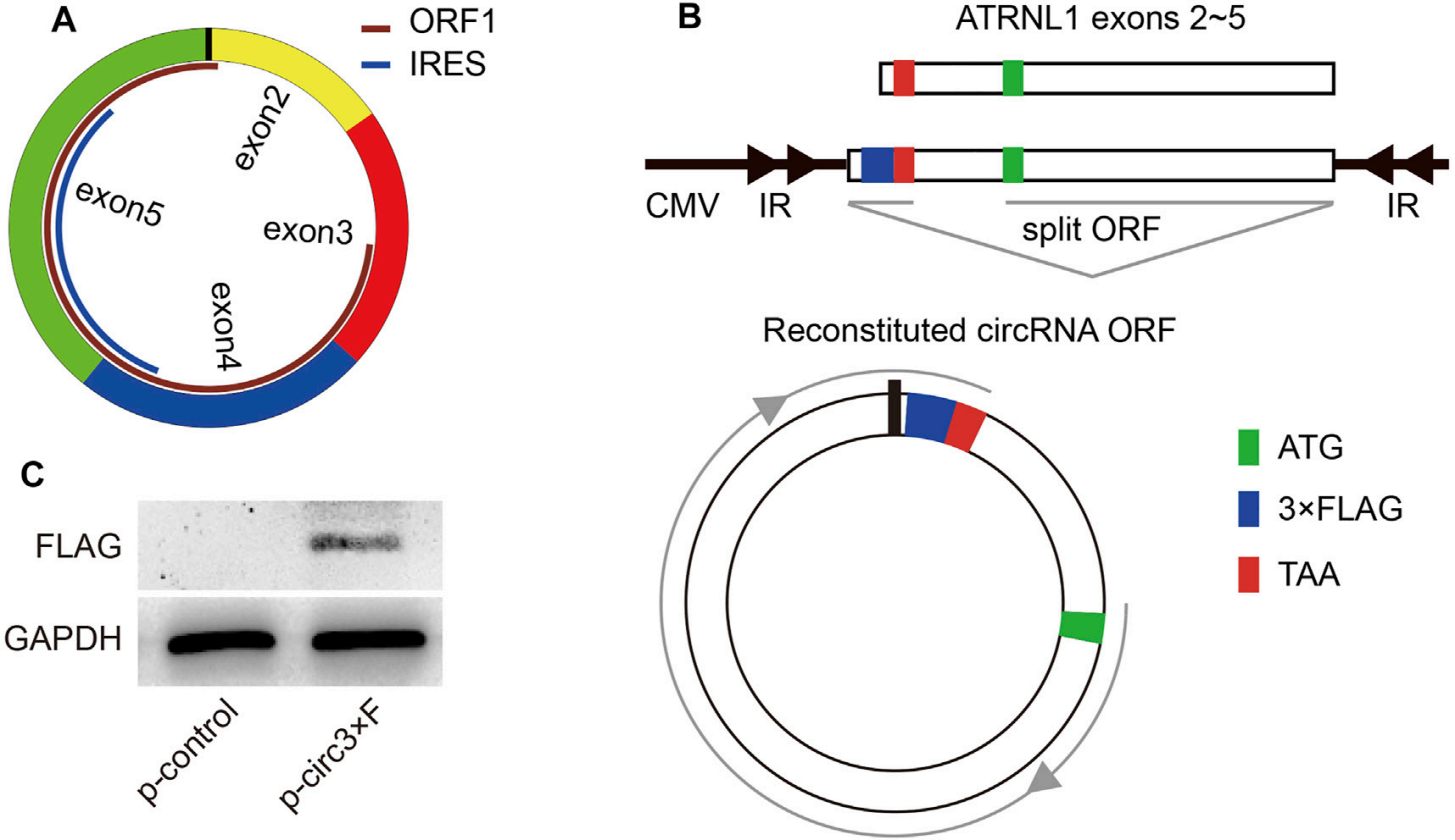
circATRNL1 encodes a protein. **(A)** circPrimer 2.0 indicates that circATRNL1 contains an open reading frame (ORF) and an internal ribosome entry site (IRES). **(B)** Schematic representation of the p-circ3×F construct. IR, inverted repeats. **(C)** Western blot analysis with an anti-FLAG antibody on proteins from cells transfected with p-circ3×F or p-control (control plasmids).

To further uncover the underlying mechanisms of circATRNL1, we explored circATRNL1 using circPrimer 2.0, which shows that circATRNL1 may encode a 131 aa protein (Figure 4A). The open reading frame (ORF) of this protein spans the splice junction of circATRNL1. An internal ribosome entry site (IRES) overlaps with the ORF and may initiate ORF translation. To test the protein-coding ability of circATRNL1, we constructed a plasmid from p-circATRNL1, named p-circ3×F, which contained a 3×FLAG-coding sequence immediately upstream of the stop codon of the ORF (Figure 4B). If the ORF has protein-coding ability, a flagged peptide will be produced. After transfecting p-circ3×F and p-control into the HEK-293 cell line for 48 h, western blotting showed that p-circ3×F produced one flagged protein (Figure 4C).

## Discussion

CircRNAs have been shown to play important roles in cancers, including ovarian cancer. However, most studies have shown that circRNAs are sponges for miRNAs. For example, hsa_circ_0015326 facilitated the progression of ovarian cancer by regulating the miR-127-3p/MYB axis (Zhang et al., 2021a). CircRAB11FIP1-induced autophagy accelerated the proliferation and invasion of epithelial ovarian cancer by binding miR-129 (Zhang et al., 2021d). Other mechanisms of circRNAs include binding proteins (Du et al., 2016; Zhang et al., 2021b), modulating the transcriptional activity of RNA Pol II (Zhang et al., 2013), coding proteins (Legnini et al., 2017; Yang et al., 2017), and competing with linear splicing (Ashwal-Fluss et al., 2014).

In the present study, we profiled the expression of circRNAs in 3 paired ovarian cancer tissues and normal ovarian tissues, and then the differentially expressed circRNAs were validated in 20 paired tissues; two circRNAs were downregulated in ovarian cancer tissues. CircZNF608 was an upregulated circRNA in RNA-seq data; however, RT–qPCR showed that it was a downregulated circRNA. The conflicting results may be due to the small sample size (*n* = 3) and intrinsic bias of RNA-Seq (Rodriguez-Esteban et al., 2015). After searching exoRBase 2.0 (www.exorbase.org) (Lai et al., 2021), we found that circZNF608 but not circATRNL1 was also expressed in exosomes from blood, urine, cerebrospinal fluid and bile. We investigated the effects of the two circRNAs on ovarian cancer cell lines and found that circATRNL1 and circZNF608 inhibited the proliferation, migration and invasion of ovarian cancer cells. These results suggested that circATRNL1 and circZNF608 may exert anticancer effects in ovarian cancer.

We investigated the underlying mechanisms by which circATRNL1 and circZNF608 exert their effects. As mentioned above, the subcellular localization of a circRNA is closely correlated with its function (Panda et al., 2017; Qu et al., 2018). CircRNAs that are mainly located in the cytoplasm may act as miRNA sponges to bind miRNAs (Qu et al., 2018). CircZNF608 and circATRNL1 are predominantly located in the cytoplasm of ovarian cancer cells, suggesting that they may exert their effects by sequestering miRNAs. Therefore, we predicted miRNA–circRNA interactions for circZNF608 and circATRNL1 and obtained 63 interactions. We validated all the interactions using a luciferase reporter assay and only obtained one positive interaction, i.e., circZNF608-miR-152-5p. A study reported that miR-152-5p was significantly downregulated in the serum/plasma of ovarian cancer patients (Langhe et al., 2015). However, we failed to find a difference in the expression level of miR-152-5p between ovarian cancer and normal ovarian tissues, suggesting that circZNF608 may competitively bind miR-152-5p but not influence its expression.

Because we failed to find an interaction for circATRNL1, we further explored the underlying mechanisms of circATRNL1 and found that circATRNL1 may exert its effects by encoding a 131 aa protein. Studies have shown that some circRNAs can be translated into functional peptides by small ORFs in a 5′ cap-independent manner (Pamudurti et al., 2017). In 2007, Legnini et al. first demonstrated that a eukaryotic endogenous circRNA encodes a protein in a splicing-dependent and cap-independent manner (Legnini et al., 2017). Yang et al. found that circ-FBXW7 contains a spanning junction ORF and encodes a novel 21-kDa protein that is driven by IRES, and circ-FBXW7 and its encoded protein have potential prognostic implications in brain cancer (Yang et al., 2018). Yang et al. reported that SHPRH-146aa encoded by circ-SHPRH is a tumor suppressor in human glioblastoma (Zhang et al., 2018a). Other coding circRNAs were also found in breast cancer (Li et al., 2020), glioblastoma (Zhang et al., 2018b; Xia et al., 2019), bladder cancer (Gu et al., 2018), colorectal cancer (Zheng et al., 2019; Zhi et al., 2019; Pan et al., 2020) and liver cancer (Liang et al., 2019). CircATRNL1 is the first reported coding circRNA in ovarian cancer. At present, seven studies have explored the roles of circATRNL1 (Zhang et al., 2019a; Wang et al., 2020a; Chen et al., 2020; Wang et al., 2021a; Wang et al., 2021b; Zhang et al., 2021c; Zheng et al., 2021), but three did not include has_circ_0020093 (Chen et al., 2020; Wang et al., 2021b; Zhang et al., 2021c). In a study by Wang et al., circATRNL1 activated Smad4 signaling and suppressed angiogenesis and ovarian cancer metastasis by binding miR-378 (Wang et al., 2021a). Zhang et al. found that circATRNL1 was downregulated in women with polycystic ovary syndrome (Zhang et al., 2019a). Zheng et al. showed that circATRNL1 is beneficial to bone marrow mesenchymal stem cell differentiation into cartilage by regulating miR-338-3p (Zheng et al., 2021). However, circATRNL1 contributes to the progression of endometriosis by promoting epithelial–mesenchymal transition (Wang et al., 2020a). Combining the findings in our study, circATRNL1 has beneficial effects on ovarian cancer and ovarian disease.

There are several limitations to our study. First, the sample size is relatively small and is therefore of limited power, which may weaken the conclusion of the present study. Second, we did not confirm the downstream effects of these two circRNAs, which may also inhibit ovarian cancer by other mechanisms. Third, circPrimer2.0 shows that circZNF608 contains an ORF and an IRES, supporting its encoding potential. However, we did not verify this finding using experiments. Fourth, SW626 may be a cell line of colonic origin (Furlong et al., 1999); thus, circATRNL1 and circZNF608 may not enact their roles specific to ovarian cancer. At least, they may also have an effect on colon cancer. Taken together, further studies based on large samples are needed to assess the expression of the 2 circRNAs, miR-152-5p and the 131 aa protein in ovarian cancer. Further studies with a functional analysis to highlight the molecular mechanisms underlying the encoded protein are proposed.

In conclusion, circATRNL1 and circZNF608 are two downregulated circRNAs in ovarian cancer and work as tumor suppressors. CircZNF608 may exert antitumor activity in ovarian cancer by binding miR-152-5p, and circATRNL1 may encode a 131 aa protein.

## Data Availability

The datasets presented in this study can be found in online repositories. The names of the repository/repositories and accession number(s) can be found below: GEO with accession GSE192410.
