# Supplementary figures and images for: CircATRNL1 and circZNF608 Inhibit Ovarian Cancer by Sequestering miR-152-5p and Encoding Protein
Source: Front Genet. 2022 Feb 23;13:784089. doi: 10.3389/fgene.2022.784089 (PMC8905624; doi:10.3389/fgene.2022.784089)

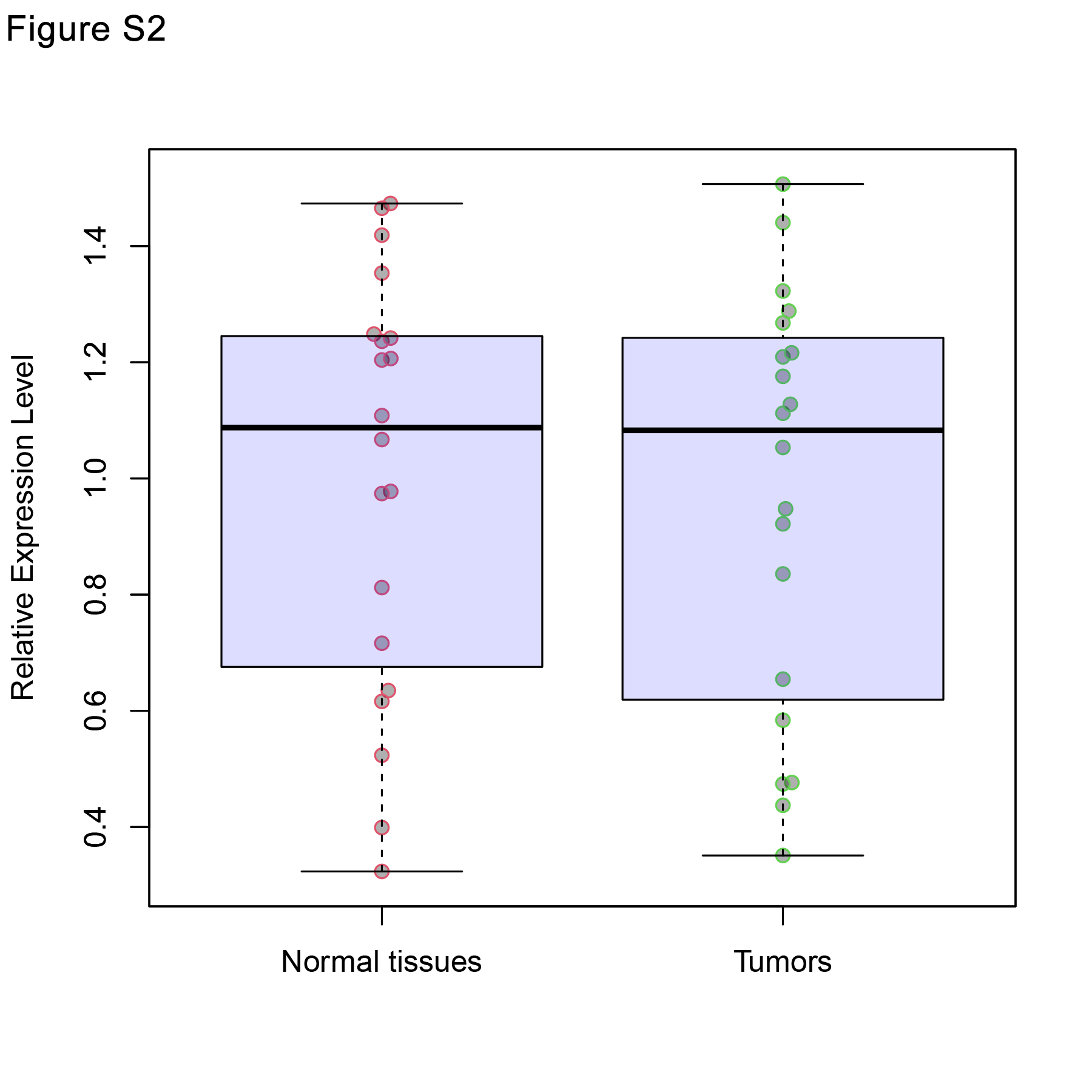

Supplement: Supplementary file 2 [file Image2.JPEG]

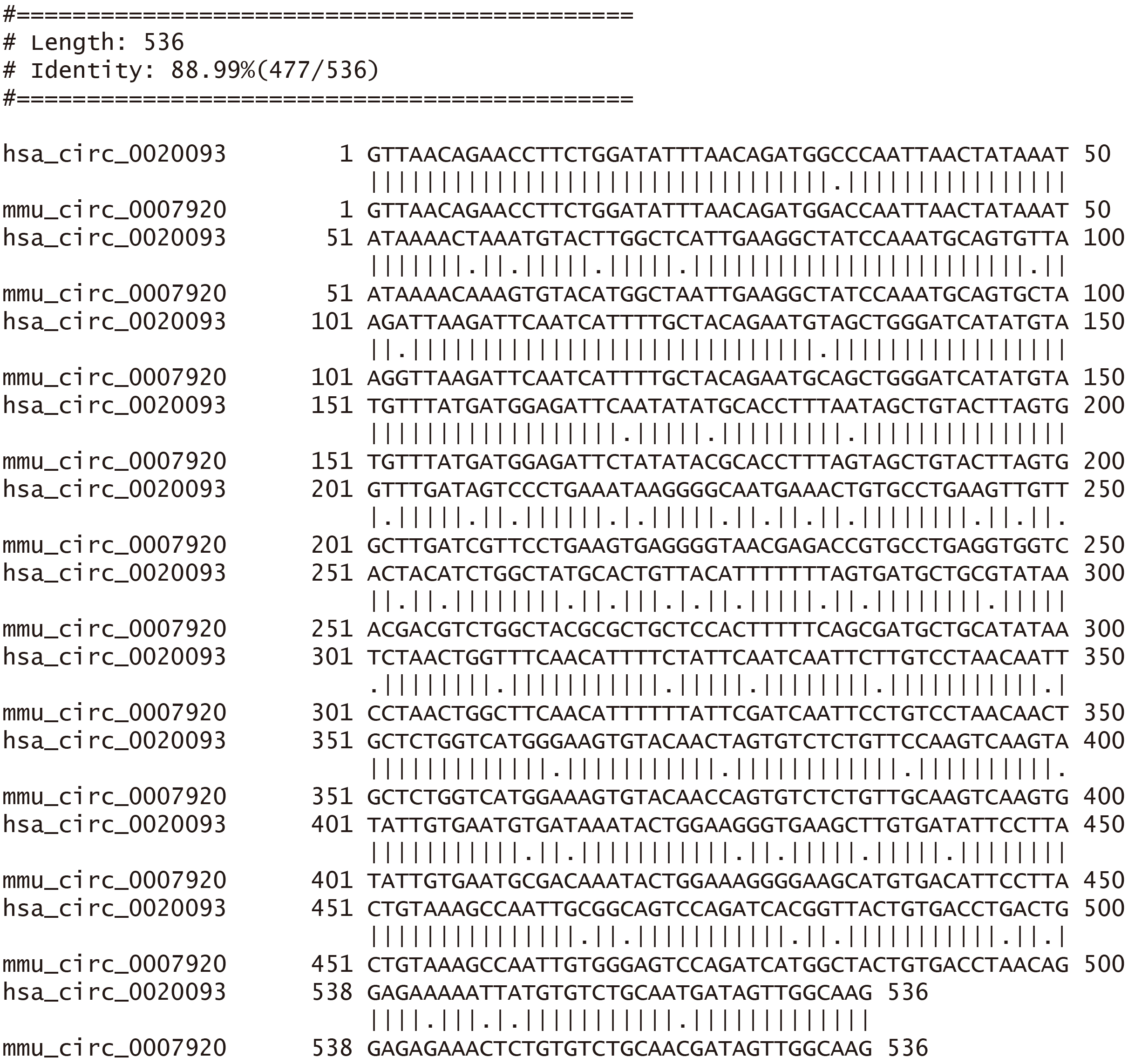

Supplement: Supplementary file 4 [file Image1.jpg]
